# *X*-Rays as an Aid to Medical Diagnosis

**Published:** 1904-11-05

**Authors:** G. Arbour Stephens


### *X*-RAYS AS AN AID TO MEDICAL DIAGNOSIS.

By G. Arbour Stephens, M.D.

The following cases may be of interest as showing how the *x*-rays can be of great service to the general practitioner, in helping him to diagnose diseases. I do not suggest that in some of them the diagnosis could not have been made without the aid of the rays, but in one or two cases they helped me to arrive at a more definite conclusion than could otherwise have been the case.

The first case was that of a young lady, who came to my consulting-room on a cold evening complaining of severe pain in her right side. Everything pointed to the disease being pleurisy, but as she was alone and very prostrate, I thought it advisable to try and do without putting her to the trouble of removing her clothes. As, however, it was necessary to make a diagnosis before she went home, I examined her with the *x*-rays and fluorescent screen, and found that the right half of the diaphragm was quite restricted in its movement, whereas the left half moved upwards and downwards to the full extent. I came to the conclusion that the only disease that could hamper the diaphragm to such an extent was pleurisy. I ordered her to take to her bed, and apply poultices. When I called the next morning I found on stethoscopic examination that there was a small patch of pleurisy at the right base, which after a short course of treatment entirely disappeared. Apart from the æsthetic side, the exertion of undressing and dressing that she was saved, undoubtedly helped her to make a good recovery.

The second case was that of a young man, who came to me in a great state of anxiety fearing that he had another attack of pleurisy. He had had one attack on the right side, and he dreaded to go through a similar experience on the other side. I examined him by means of the *x* rays and screen, and found that both halves of the diaphragm moved well and equally, and with this information I assured him that he need have no fear of another attack of pleurisy. In two days' time he reported himself as quite well and entirely free from pain.

The third case was that of a young man, who had been condemned to exile in South Africa, on account of what his doctor in the country had told him was "consumption." On examination with the *x*-rays I found both lungs quite transparent and clear. The stethoscopic examination was not very definite. I told him that I considered he was free from tubercular disease, and that I did not consider it necessary for him to go to South Africa. In two months' time he reported himself quite well, and heavier by over a stone.

The fourth case was that of a married lady, aged 34, who had been told by two South of England doctors "that she was consumptive." As a result she had been very worried, and lost weight very considerably. Stethoscopic examination was in this case also very indefinite, but on examination with the *x*-rays the lungs appeared to be quite healthy. As a result of this information she immediately brightened, and in a short time put on several pounds in weight. The last report I had of her was that she was quite well.

The fifth case was an elderly gentleman, about 62, who complained of marked shortness of breath on going up even the least incline. As he was very thin, I thought it would be interesting to examine him with the *x*-rays, and, as a result, I could see the heart beating irregularly.

The sixth case was a young man, who came to my room very carefully wrapped and muffled up. He stated that he was anxious for me to examine him. After a careful stethoscopic examination I could find nothing wrong with his lungs, so I decided to complete the examination by looking at him with the $x$-rays. These rays only confirmed the stethoscopic result, and I informed him that I could find nothing wrong with him. Having expressed relief at such a diagnosis, he told me he had just got out of bed, after being treated there during six weeks for pleurisy.

Greater accuracy in diagnosis considerably increases one's interest in a case, to say nothing of the gain from a patient's point of view.

